# Supplementary material for: Aerodynamic forces and flows of the full and partial clap-fling motions in insects
Source: PeerJ. 2017 Mar 9;5:e3002. doi: 10.7717/peerj.3002 (PMC5346288; doi:10.7717/peerj.3002)
Supplement: Supplemental Information 1 [file peerj-05-3002-s001.doc]

**Electronic Supplemental Material to “Aerodynamic forces and flows of the full and partial clap-fling motions in insects”**

Xin Cheng* and Mao Sun

Institute of Fluid Mechanics, Beijing University of Aeronautics & Astronautics, Beijing 100191, China (*x.cheng@buaa.edu.cn)

**S1 Solution method of the Navier-Stokes equations**

The computational method is the same as that used by Sun and Yu^1^ and Cheng and Sun^2^ and only an outline of it is given here. The method is based on the artificial-compressibility algorithm, which was first developed by Rogers et al.^3^ for single-grid, and then extended by Rogers and Pulliam^4^ to overset grids. In the method, the time derivatives of the momentum equations were differenced using a second-order, three-point backward difference formula. To solve the time discretized momentum equations for a divergence free velocity at a new time level, a pseudo-time level is introduced into the equations and a pseudo-time derivative of pressure divided by an artificial compressibility constant was introduced into the continuity equation. The resulting system of equations were iterated in pseudo-time until the pseudo-time derivative of pressure approaches zero, thus, the divergence of the velocity at the new time level approached zero. The derivatives of the viscous fluxes in the momentum equation were approximated using second-order central differences. For the derivatives of convective fluxes, upwind differencing based on the flux-difference splitting technique was used. A third-order upwind differencing was used at the interior points and a second-order upwind differencing used at points next to boundaries. With overset grids, for each wing there was a body-fitted curvilinear grid, which extends a relatively short distance from the wing, and in addition, there was a background Cartesian grid, which extends to the far-field boundary of the domain. The solution method for single-grid was applied to each of these grids; data were interpolated from one grid to another at the inter-grid boundary points. Details of this algorithm can be found in Refs. [3,4]. For far field boundary conditions, at the inflow boundary, the velocity components were specified as free-stream conditions while pressure was extrapolated from the interior; at the outflow boundary, pressure was set equal to the free-stream static pressure and the velocity was extrapolated from the interior. On the wing surfaces, impermeable wall and no-slip boundary conditions were applied, and the pressure on the boundary was obtained through the normal component of the momentum equation written in the moving coordinate system. The wing grids were generated using a Poisson solver which was based on the work of Hilgenstock^5^; they are of O-H type grids. The background Cartesian grid was generated algebraically.

The moving overset-grid solver used in the present study was developed by Sun and Lan^6^ and Sun and Yu^1^. It was tested by comparison with the analytical solution of the starting flow around a sphere and with the measured forces on a flapping model fruit fly wing^6^. In the studies on clap-fling mechanism by Sun and Yu^1,7^, the solver was further tested by the experimental data of Spedding and Maxworthy^8^, for a wing-pair performing clap-fling motion. Flow visualization and lift measurement data for the fling motion are available in Ref. 8. Figure 5 of Ref. 7 compared the calculated and experimental streamline patterns for various opening angles; Figure 5 of Ref. 1 compared the calculated and experimental lift as a function of time. It is seen that the calculated flow field and lift are in reasonably good agreement with the experimental results.

Before proceeding to compute the flows, grid resolution tests were conducted to ensure that the flow calculation was grid independent. For a clear description of the time courses of the forces, we express the time during a cycle as a non-dimensional parameter, , such that =0 at the start of an upstroke, and =1 at the end of the subsequent downstroke. Three grid-systems were considered. For grid-system 1, the wing grid had dimensions 416143 around the wing, in the normal direction and in the spanwise direction, respectively (first layer grid thickness was 0.0015*c* where *c* is the mean chord length of wing), and the background grid had dimensions 818181 in the *x*_E_, *y*_E_ and *z*_E_ directions, respectively. For grid-system 2, the corresponding grid dimensions were 619165 and 121121121 (first layer grid thickness was 0.001*c*). For grid-system 3, the corresponding grid dimensions were 9113596 and 181181181 (first layer grid thickness was 0.00067*c*). In all the grid-systems, the outer boundary of the wing-grid was set at about 2.5*c* from the wing surface and that of the background-grid at 20*c* from the wings. For all the three grid-systems, grid points of the background grid concentrated in the near field of the wings where its grid density was approximately the same as that of the outer part of the wing-grid. Figure S1 shows the computed lift (*C_L_*) and drag (*C_D_*) coefficients of a fly (VL1) in one cycle; results calculated with three grid-systems are plotted. Figure S2(a) shows the contours of the non-dimensional spanwise component of vorticity at half-wing length at =0.75 and Figure S2(b) shows the corresponding iso-vorticity surface.


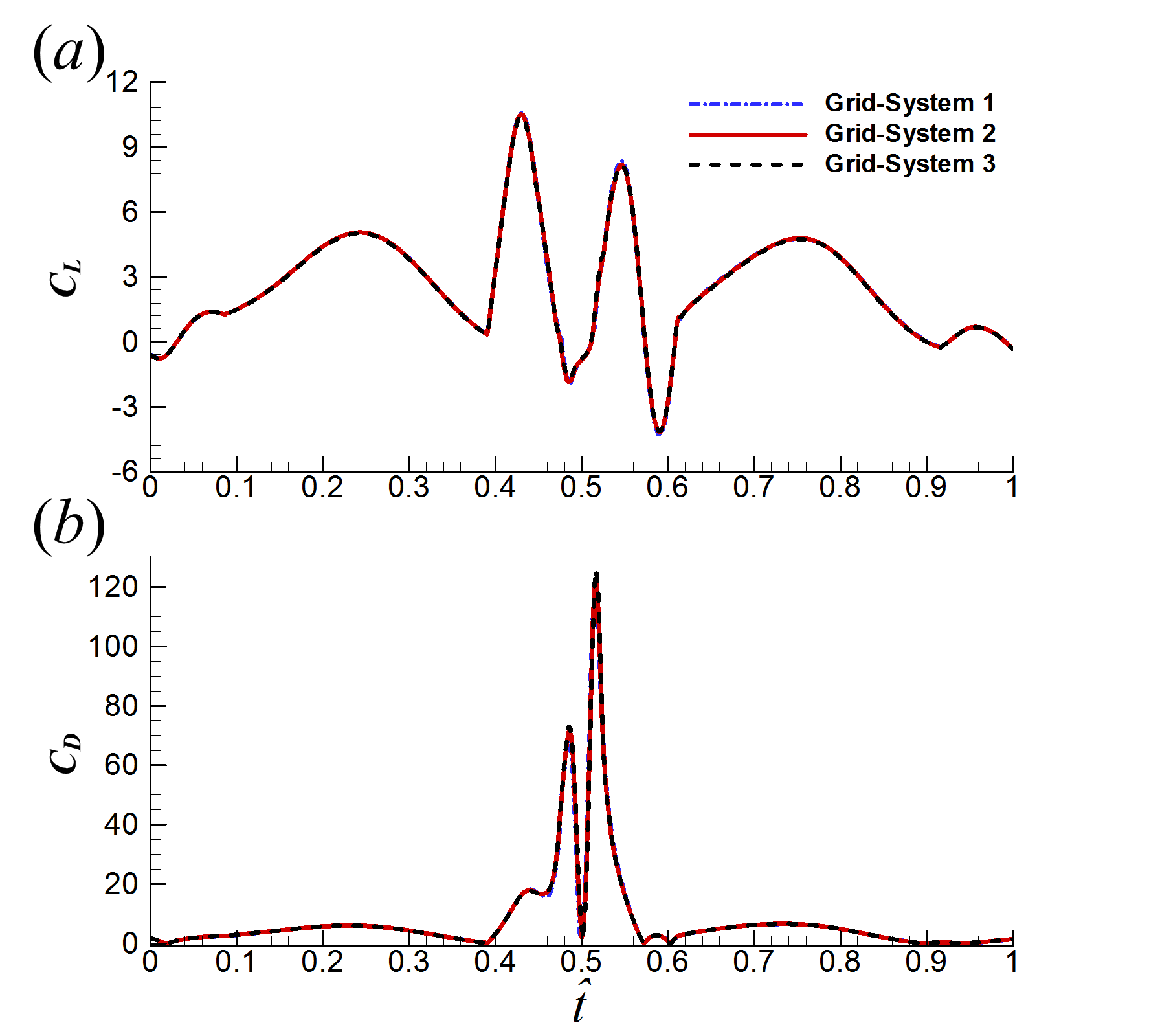


Figure S1. Time courses of the *C_L_* (a) and *C_D_* (b) in one cycle, calculated with three grid-systems.


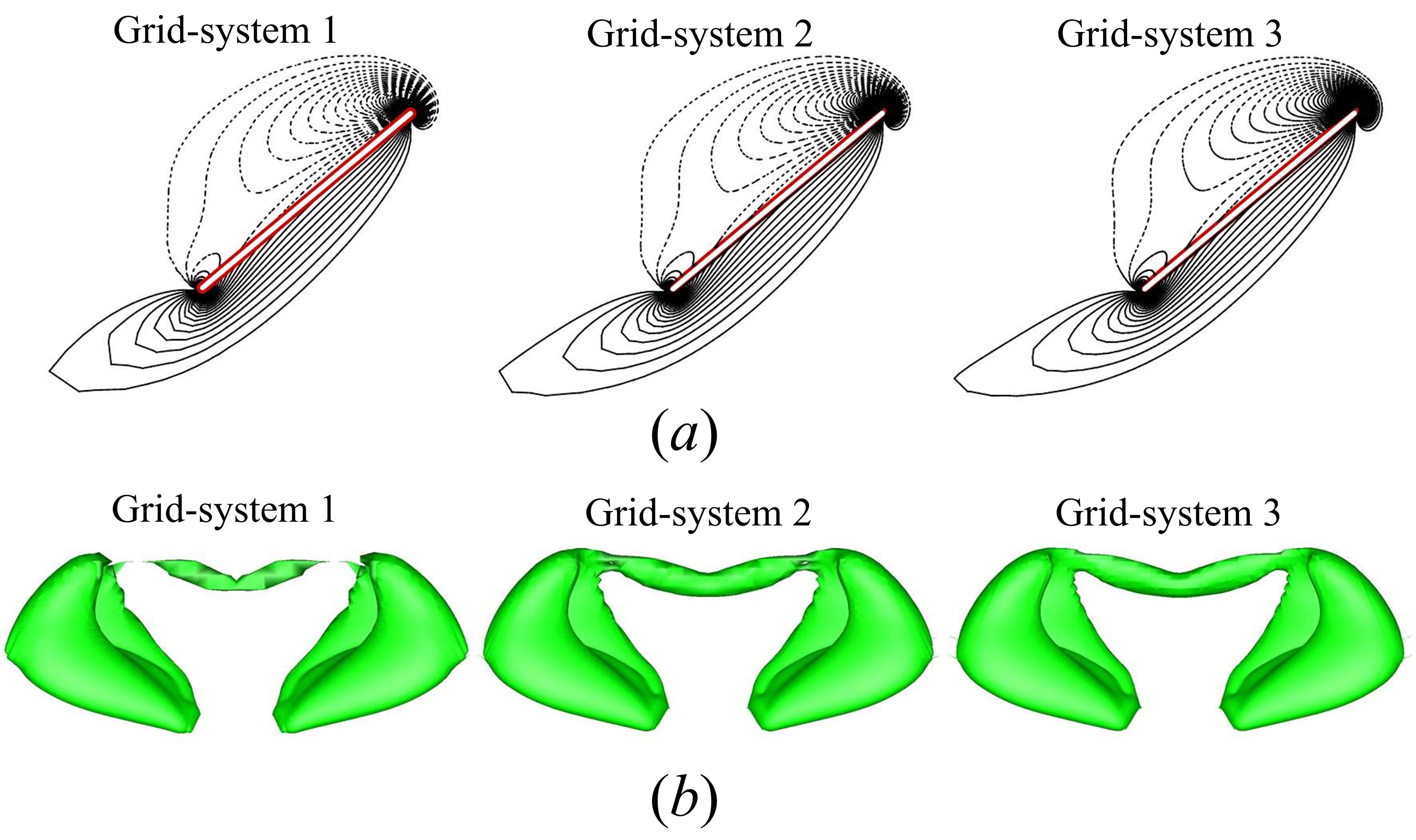


Figure S2. Vorticity fields computed with different grid-systems. (a) Contours of non-dimensional spanwise component of vorticity at half-wing length (the magnitude of the vorticity at the outer contour is 2 and the contour interval is 1). (b) Iso-vorticity surface plots (the magnitude of the non-dimensional vorticity is 3).

It is observed that the first grid refinement produces a little change in the vorticity plot, and the second grid refinement produces almost no change (Figure S2). There is almost no difference between the force coefficients calculated by the three grid-systems (Figure S1). Calculations were also conducted using a larger computational domain. The domain was enlarged by adding more grid points to the outside of the background grid of grid-system 2. The calculated results showed that there was no need to put the outer boundary further than that of grid-system 2. The non-dimensional time step was 0.02 (non-dimensionalized by *c*/*U* where *U* is mean velocity of the wing at the radius of the second moment of wing area). The effect of time step value was studied and it was found that a numerical solution effectively independent of the time step was achieved if the time step value was ≤0.02. From the above discussion, it was concluded that grid-system 2 and time step was 0.02 were proper for the calculation.

**S2 Calculations in which a sinusoidal *α* variation is employed**

In this study, *α* is assumed to be constant in the translation phase and varies only at stroke reversal. It is of interest to see how will the obtained results vary with a different *α* variation. A sinusoidal variation of *α* is employed in the FCF, PCF and SW cases and the wing root distance is enlarged in the two-winged cases to avoid physical touch between wings (the closest distance between wings (*d*) remains unchanged compared to the cases in the main text). The computed results are shown in Figure S3 (the results are discussed in the main text).


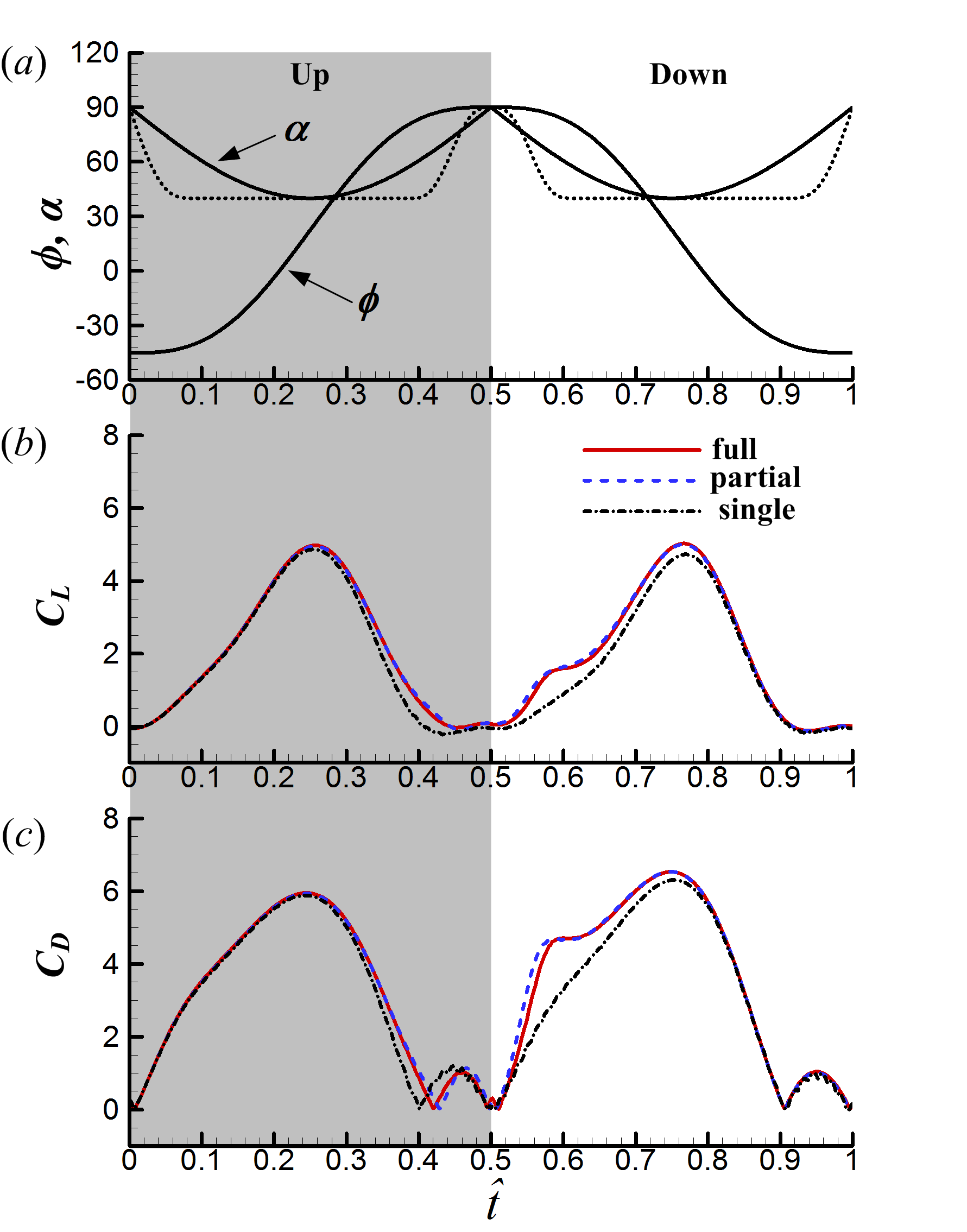


Figure S3. Time courses of the wing motion and force coefficients in one cycle for the three different cases: (a) positional angle (*φ*) and angle of attack (*α*) in one cycle (dashed line represents the initial *α* variation); (b) the lift coefficient; (c) the drag force coefficient.

**S3 Calculations in which other partial clap-fling motions are considered**

The sensitivity analysis of gap distance between wings in full clap-fling motion was studied by Sun and Yu^1^ and the sensitivity analysis of angular excursion in partial clap-fling motion can be seen in Lehmann et al.’s work^9^. In order to show the effect of wing root distance and angular excursion on the performance of partial clap-fling motion, we made some additional computations in which some other combinations of root distance and angular excursion are considered. A new variable, the angular distance *θ* between the spanwise axis of each wing and the mid plane at $\hat{t}$=0.5 (the end of the clap phase), is introduced to characterize different motions (Fig. S4a). In all the clap-fling motions, the distance between wing tips at $\hat{t}$=0.5 is kept constant at *d*=0.1*c*. By this definition, the FCF and PCF cases studied correspond to the *θ*=0° case and *θ*=7.9° case respectively. Four other partial clap-fling motions correspond to the *θ*=2° case, *θ*=4° case, *θ*=6° case and *θ*=10° respectively. The $\bar{C}_{L}$, $\bar{C}_{D}$ and $\bar{C}_{L}/\bar{C}_{D}$ of all the six cases are shown in Figure S4 (for comparison, the values of $\bar{C}_{L}$, $\bar{C}_{D}$ and $\bar{C}_{L}/\bar{C}_{D}$ of the SW case are indicated as horizontal dashed lines in Fig. S4b-d respectively). The highest $\bar{C}_{L}$ enhancement is obtained in the *θ*=0° case (FCF), but to do so a much large $\bar{C}_{D}$ must be overcome (i.e. at much larger energy consumption), resulting in a very inefficient $\bar{C}_{L}/\bar{C}_{D}$ value. However, if *θ* is slightly increased to *θ*=2°, one can have a substantial $\bar{C}_{L}$ augmentation without a large $\bar{C}_{D}$ ($\bar{C}_{L}$ is increased by 21% and $\bar{C}_{L}/\bar{C}_{D}$ is just a hair lower than that of the SW case). When *θ* is further increased, $\bar{C}_{L}$ and $\bar{C}_{D}$ continue to decrease gradually while $\bar{C}_{L}/\bar{C}_{D}$ stays almost constant. It is suggested that the two wings should be close enough, but not too close, to have a good interference effect.


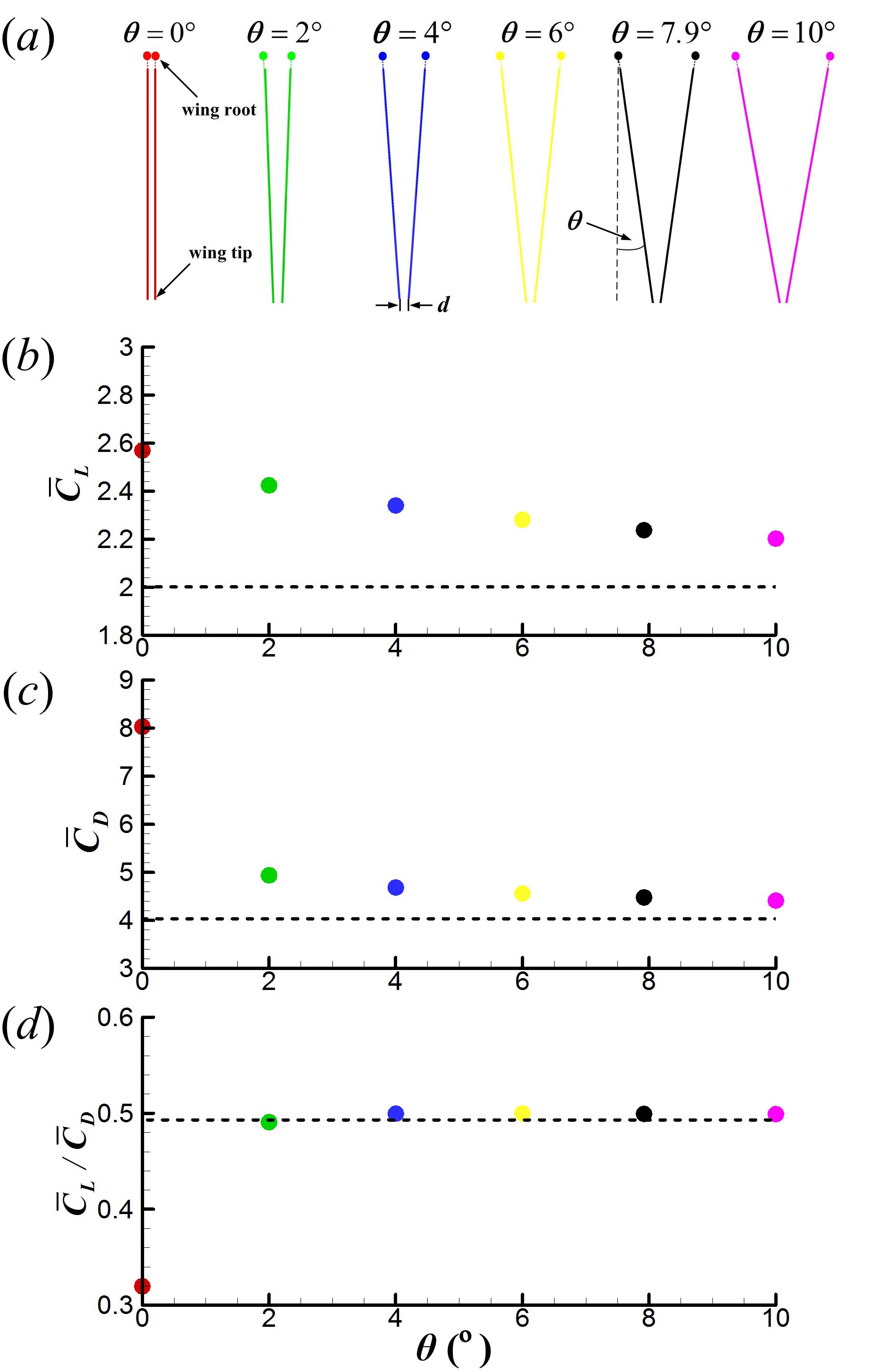


Figure S4. Aerodynamic force performance of different clap-fling motions. (a) All motions characterized by different angular distances (*θ*) (the FCF and PCF cases studied above correspond to the *θ*=0° case and *θ*=7.9° case respectively). (b) Mean lift coefficient. (c) Mean drag coefficient. (d) Lift-to-drag ratio. The horizontal dashed lines in (b)-(d) indicate the values of $\bar{C}_{L}$, $\bar{C}_{D}$ and $\bar{C}_{L}/\bar{C}_{D}$ of SW case respectively.

**References**

1. Sun, M. & Yu, X. Aerodynamic Force Generation in Hovering Flight in a Tiny Insect. *AIAA J.* **44,** 1532–1540 (2006).

2. Cheng, X. & Sun, M. Wing-kinematics measurement and aerodynamics in a small insect in hovering flight. *Sci. Rep.* **6,** 25706 (2016).

3. Rogers, S. E., Kwak, D. & Kiris, C. Steady and unsteady solutions of the incompressible Navier-Stokes equations. *AIAA J.* **29,** 603–610 (1991).

4. Rogers, S. & Pulliam, T. Accuracy enhancements for overset grids using a defect correction approach. in *32nd Aerospace Sciences Meeting and Exhibit* (American Institute of Aeronautics and Astronautics, 1994).

5. Hilgenstock, A. A fast method for the elliptic generation of three dimensional grids with full boundary control. in Num. Grid Generation in CFM’88 (eds Sengupta, S. et al.). 137-146 (Ld, 1988).

6. Sun, M. & Lan, S. L. A computational study of the aerodynamic forces and power requirements of dragonfly (Aeschna juncea) hovering. *J. Exp. Biol.* **207,** 1887–1901 (2004).

7. Sun, M. & Yu, X. Flows around two airfoils performing fling and subsequent translation and translation and subsequent clap. *Acta Mech. Sin.* **19,** 103–117 (2003).

8. Spedding, G. R. & Maxworthy, T. The generation of circulation and lift in a rigid two-dimensional fling. *J. Fluid Mech.* **165,** 247 (1986).

9. Miller, L. A. & Peskin, C. S. A computational fluid dynamics of `clap and fling’ in the smallest insects. *J. Exp. Biol.* **208,** 195–212 (2005).
